# Attenuated inferred–sensory mismatch during masked face recognition in schizophrenia

**DOI:** 10.3389/fpsyt.2026.1749435

**Published:** 2026-03-16

**Authors:** Masakazu Sunaga, Yuichi Takei, Yutaka Kato, Reiko Oi, Kohei Imai, Keisuke Fukasawa, Yoshihito Shigihara, Kazuyuki Fujihara, Tatsuki Sekiya, Masato Fukuda, Seiichiro Jinde

**Affiliations:** 1 Department of Psychiatry and Neuroscience, Gunma University Graduate School of Medicine, Gunma, Maebashi, Japan; 2 Tsutsuji Mental Hospital, Gunma, Tatebayashi, Japan; 3 Gunma Prefectural Psychiatric Medical Center, Gunma, Isesaki, Japan; 4 Kumagaya General Hospital, Saitama, Japan

**Keywords:** Bayesian brain hypothesis, face recognition, insular cortex, magnetoencephalography, predictive processing, schizophrenia

## Abstract

**Introduction:**

Predictive-processing accounts have become increasingly influential in understanding the pathophysiology of schizophrenia (SZ). Within this framework, mismatch negativity (MMN) paradigms provide a well-established and widely replicated assay of mismatch processing. However, most MMN designs operationalize mismatch as violations of externally defined regularities. In this study, we aimed to use magnetoencephalography and a masked-face paradigm to probe a complementary form of mismatch that arises when an internally inferred representation of an occluded face is confronted with newly available sensory evidence after unmasking (an inferred–sensory mismatch).

**Methods:**

Participants first viewed the masked faces, followed by the same faces without masks. Two types of stimuli—individual and average—were used. Data from 18 individuals with SZ and 18 healthy controls (HCs) were analyzed using spatiotemporal cluster tests.

**Results:**

Among HCs, mask removal from individual faces elicited a significant cluster at the left temporal sensors (234–511 ms), with source estimates suggesting involvement of the left insular cortex. No significant clusters were observed for average faces. In the SZ group, no significant mismatch responses were observed in either condition.

**Discussion:**

Overall, HCs showed a reliable neural response to the unmasking of individual faces, consistent with a mismatch between an internally inferred representation and newly revealed sensory information. In contrast, no robust mismatch-related response was detected in the SZ group in the present dataset. This pattern is compatible with predictive-processing accounts that emphasize reduced precision of perceptual inference in SZ.

## 1 Introduction

Computational psychiatry is a promising framework for understanding psychiatric disorders using mathematical models. Schizophrenia (SZ) has been a key focus of predictive-processing accounts (1–3). Within this broader theoretical context, we proposed the Hyperlearning Hypothesis, in which abnormalities in prediction-related processing are conceptualized in SZ as the initiating conditions for maladaptive overlearning, rather than as stable or sufficient causes of the disorder (4). In this framework, imprecise and distorted prediction-error signals are posited as a proximal trigger that repeatedly drives learning mechanisms, thereby promoting excessive reinforcement of inaccurate internal models and, over time, contributing to progressive network-level reorganization. This perspective builds on and reinterprets the Bayesian brain hypothesis, which posits that perception and cognition emerge from the formation and updating of internal models based on predictions and prediction errors (5–8). A critical investigation of this framework is whether such abnormalities arise when observers must infer hidden information from incomplete sensory input. From a Bayesian viewpoint, the brain continuously compares incoming sensory input with prior expectations, registering mismatches when predictions fail. For example, when an earthquake suddenly shakes a building, the brain registers a prediction error or “surprise” from the mismatch between the expected and actual sensory input. These errors are minimized by updating internal models in the brain, thereby adjusting to unexpected changes (9). This predictive updating may underlie diverse perceptual and cognitive processes.

Generally, prediction errors are computed by comparing sensory inputs with prior expectations, and predictions are updated accordingly. However, according to the Bayesian brain hypothesis, the precision of prediction, sensory input, and prediction errors decreases in SZ (1, 10). This leads to faulty internal model updating, resulting in “false inference,” which may underlie symptoms such as hallucinations and delusions (1, 10). Computational models indicate that reduced precision contributes to various symptoms, including hallucinations, delusions of control, catatonia, and attenuated mismatch negativity (MMN) (1). Accordingly, predictive and inferential processes in SZ have been experimentally assessed in an increasing number of studies, and this framework has attracted growing attention (11).

Crucially, from this Bayesian perspective, perception reflects the integration of prior expectations with sensory evidence, and prediction errors arise when new evidence forces an update of an inferred representation—processes likely compromised in SZ owing to reduced precision (1, 10). A well-established experimental readout of such predictive processing in SZ is the MMN, an event-related potential elicited by deviant auditory stimuli in a stream of repeated standard tones (12–15). It reflects the automatic detection of deviance and is interpreted as evidence of predictive processing in the auditory system (16–18). Its amplitude is consistently reduced in SZ (12, 19, 20). However, conventional MMN paradigms typically rely on short-term regularities in simple, non-social sounds and define deviance primarily in terms of externally specified stimulus features (21, 22). Therefore, these paradigms offer limited emphasis on situations in which observers must infer the most likely cause of incomplete sensory input and update this inferred representation when new evidence becomes available. Paradigms that incorporate socially relevant stimuli and explicitly engage such inference may therefore complement conventional MMN approaches.

Face recognition is a core aspect of visual social cognition and relies heavily on prior experience and learning (23). When presented with incomplete facial information, observers tend to perceptually complete missing parts using internalized priors—for example, by filling in features toward an “average” face representation (24). Consistent with this observation, occluding the lower face, such as wearing a mask, can shift perceived appearance toward average-related impressions, including perceived attractiveness (25, 26). For example, electrophysiological studies have revealed smaller N170 face-selective responses to average and prototypical faces, which is consistent with the notion that observers rely on an internal average face template during face perception (27, 28). More generally, in perceptual inference, the brain may construct internal representations of objects or scenes based on incomplete or ambiguous sensory input (5–8). However, when additional sensory evidence becomes available, the newly revealed input may not fully align with the previously inferred representation. The resulting discrepancy reflects a mismatch between an internally inferred representation and later sensory evidence at the descriptive level, without committing to any specific computational interpretation, such as explicit signaling of prediction error or updating of a learning-based model. We refer to this class of discrepancies as inferred–sensory mismatch (ISM), defined as a divergence between a representation inferred under conditions of uncertainty and the sensory evidence that later constrains or revises that representation. This paradigm provides a natural context for examining neural responses associated with ISM.

The primary aim of this study was to operationalize belief updating under uncertainty in a socially meaningful context by quantifying neural responses to ISM in a masked-face paradigm. We subsequently examined whether this differentiation between masked-to-unmasked transitions and initially unmasked presentations differs in SZ. In this paradigm, masked-face perception provides a concrete and ecologically relevant example; observers infer missing facial features under occlusion, and mask removal reveals sensory information that may partially diverge from this inferred representation. Building on this rationale, we formulated the following hypothesis in SZ: neural responses reflecting ISM would show reduced differentiation between masked-to-unmasked transitions, which engage inference from incomplete facial input, and initially unmasked presentations, which reflect ordinary face recognition. Notably, whether such differentiation is robustly present in healthy controls (HCs) under our paradigm was not known *a priori*. Therefore, we initially assessed within-group differences to establish the presence and direction of this differentiation in HCs, and subsequently examined whether the same pattern of differentiation was attenuated in SZ. Additionally, we performed between-group comparisons of this differentiation and used magnetoencephalography (MEG) to quantify ISM in this paradigm.

## 2 Materials and methods

### 2.1 Participants

We recruited 19 patients with SZ and 20 HCs between May 2023 and September 2024 from Gunma Prefectural Psychiatric Medical Center, Tsutsuji Mental Hospital, and Gunma University Hospital, Japan. This study was reviewed and approved by the Ethics Committee of Gunma University Graduate School of Medicine, Japan, and was registered at the University Hospital Medical Information Network Clinical Trials Registry (ID: UMIN000050008). Although the preregistration outlined an aspirational target sample size, data acquisition could not be continued beyond September 2024 because MEG measurements became operationally infeasible at our site owing to constraints related to helium costs. Consequently, recruitment was stopped earlier than initially planned, and the final sample reflects all eligible participants enrolled during the measurement period. Planned connectivity analyses mentioned in the preregistration are not reported here to maintain a focused scope and will be addressed in future work.

All participants provided written informed consent after receiving a detailed study explanation. This study was conducted following the Declaration of Helsinki guidelines. Handedness was assessed using the Edinburgh Handedness Inventory to confirm that all registered participants were right-handed (29). SZ diagnoses were confirmed using the Structured Clinical Interview for DSM-5 Research Version (30), and HCs were screened using the same interview to exclude any current or past psychiatric disorders. HCs were also screened to exclude severe physical comorbidities. The exclusion criteria are summarized in **Supplementary Material 1**. Psychopathology was assessed using the Positive and Negative Syndrome Scale (PANSS) (31), Japanese Adult Reading Test (JART; a brief measure of intelligence quotient [IQ]) (32), and the Global Assessment of Functioning (GAF) scale (33). Chlorpromazine equivalent doses of antipsychotics were calculated for participants with SZ (34). Disease duration was determined as the difference between the age at study participation and SZ onset.

One patient with SZ was excluded for ambidexterity. One HC was excluded because of an incidental brain tumor, and another owing to a psychiatric disorder identified during screening. Consequently, data from 18 patients with SZ and 18 HCs were included in the final analysis.

Group differences in sex distribution were assessed using the chi-square test. Age, JART scores, and GAF scores, as well as the accuracy and response times in the experimental tasks, were compared between the groups using Welch’s t-test (**Table 1**).

**Table 1:** Participant characteristics.

	HC (n = 18)		SZ (n = 18)		χ^2^	*p*-value
	M	F	M	F		
Sex	9	9	5	13	1.052	0.305
	Mean	SD	Mean	SD	t	*p*-value
Age (year)	45.0	12.0	38.7	11.4	-1.627	0.113
JART	111.9	7.7	103.0	9.5	-3.109	0.004*
GAF	80.9	7.5	63.4	9.3	-6.177	0.000*
Disease duration (year)	–	–	12.4	10.1	–	–
PANSS positive symptoms	–	–	15.4	4.4	–	–
PANSS negative symptoms	–	–	16.8	4.5	–	–
PANSS general psychopathology	–	–	37.0	9.6	–	–
Antipsychotic (chlorpromazine equivalent dose mg/day)	–	–	504.3	317.8	–	–

*Statistical significance: p < 0.05.

GAF, Global Assessment of Functioning; HC, healthy control; JART, Japanese Adult Reading Test; PANSS, Positive and Negative Syndrome Scale; SZ, schizophrenia; SD, standard deviation.M, Male; F, Female.

The two groups did not significantly differ in age or sex. The mean illness duration in the SZ group was 12.4 years, indicating that the cohort primarily comprised chronic outpatients. The SZ group showed significantly lower GAF scores and IQ, as estimated using the JART, than the HC group; however, the participants demonstrated adequate understanding and performance of the experimental tasks.

### 2.2 Stimuli and procedure

**Figure 1:**
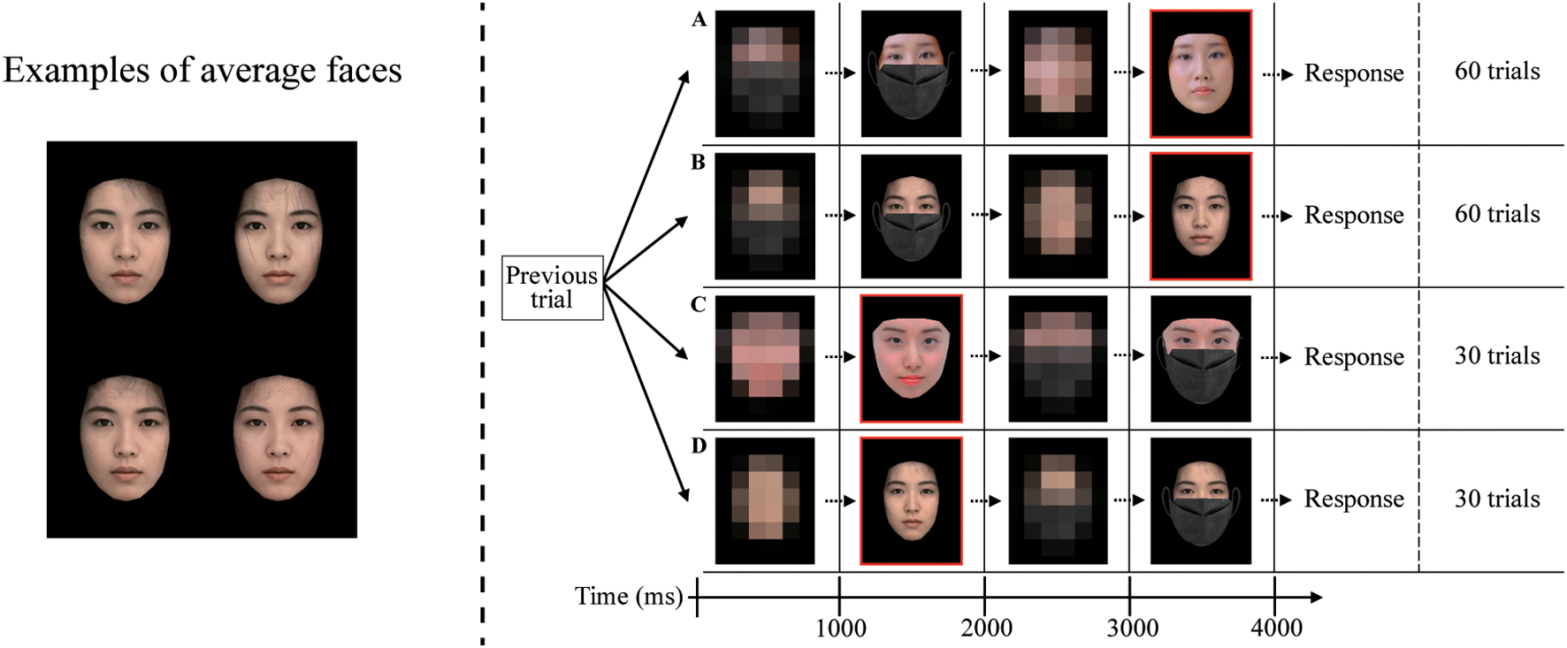
Schematic of the experimental procedures. The left panel shows sample face images used in the actual experiment. The right panel shows the sequence of events for each trial. Following a random order from the previous trial, one of the four trial types was presented: **(A)** a masked individual face followed by its unmasked version (*Forward-order unmasking*, 60 trials), **(B)** a masked average face followed by its unmasked version (*Forward-order unmasking*, 60 trials), **(C)** an unmasked individual face followed by its masked version (*Reverse-order control*, 30 trials), and **(D)** an unmasked average face followed by its masked version (*Reverse-order control*, 30 trials). At the end of each trial, the participants were asked whether the two faces represented the same identity. Participants responded by pressing a button. The next trial commenced immediately after the response. To eliminate the effects of luminance changes, a pixelated version of the face was presented as a pre-stimulus before each facial image. The red frame indicates the epochs that were used for the actual data analysis. Source: Mask overlay designed by rawpixel.com, https://www.freepik.com/free-photo/black-protective-fabric-face-mask_13299938.htm, reproduced under Free license. Face images created by yslab, https://en.photo-ac.com/photo/24800977 and TicTac, https://en.photo-ac.com/photo/3167326, reproduced under photoAC's Standard License.

Individual facial images of women with neutral expressions were used as experimental stimuli. Only female faces were used because, among the available photographs, only a set of female faces met our criteria (neutral expression and consistent acquisition conditions). To eliminate the influence of hair and background color, Facemorpher (35) was used to extract only the facial region, and the background was replaced with black. A mask was then applied to each image to obscure the lower half of the face. Additionally, we created average morphed faces by randomly combining the faces of 10 individuals using WebMorph (36). The lower halves of the average faces were covered with a mask. An average face contained fewer distinctive features than the individual faces; nevertheless, each average face remained visually distinguishable (**Figure 1**).

**Figure 1** illustrates the experimental procedure. To minimize the influence of abrupt luminance changes on MEG measurements, we inserted a pixelated (mosaic) version of the face before each face presentation. Accordingly, each trial consisted of an initial mosaic display (1000 ms), a face stimulus (1000 ms), a second mosaic display (1000 ms), and the corresponding face stimulus (1000 ms), resulting in a fixed trial duration of 4000 ms. Participants indicated whether the two successive face images represented the same identity by pressing a button. They were allowed unlimited time to respond, and the next trial began only after a response was recorded.

The task included two randomly intermixed trial types: *Forward-order unmasking*, in which a masked face was presented first and subsequently the corresponding unmasked face, and *Reverse-order control*, in which an unmasked face was presented first and then the masked version. The *Reverse-order control* trials were included to measure neural responses during ordinary face recognition. Each trial type included two face-types: individual faces and average faces. For *Forward-order unmasking*, each face type comprised 60 trials. For analysis, we used 50 trials per face type in which the masked and unmasked faces depicted the same identity. Across the analyzed trials, 25 unique face identities were prepared for each face type (individual and average), and each identity appeared twice in a randomized order. As the number of eligible face identities was limited, this repetition was necessary to obtain a sufficient number of trials for stable estimation of event-related fields (ERFs). For *Reverse-order control*, each face type comprised 30 trials to reduce participant burden. For these trials, 30 unique face identities were prepared per face type, and each identity was presented once in a randomized order. The experimental session lasted approximately 30 min including preparation and instructions, and the task completion required approximately 15–20 min.

### 2.3 MEG data acquisition and preprocessing

Brain activity during task performance was recorded using a whole-head MEG system (RICOH160-1; RICOH Co., Ltd., Tokyo, Japan) equipped with 160-channel axial gradiometers. Recordings were conducted in a magnetically shielded room at the Kumagaya General Hospital. Participants in the supine position were instructed to remain awake and relaxed throughout the recording. The gradiometer sensor coils measured 15 mm in diameter and 50 mm in height, with paired coils separated by 23 mm. Data were sampled at 2,000 Hz and filtered online with a 500 Hz low-pass filter. For the co-registration of MEG data with anatomical brain images, five fiducial magnetic marker coils were attached to the participant’s face: one located 40 mm above the nasion, two bilaterally 10 mm anterior to the tragus, and two at the bilateral preauricular points. The spatial coordinates of these markers were digitized immediately before MEG recording. To ensure optimal alertness, recording began immediately after the magnetically shielded room door was closed. Trained medical technicians continuously monitored the participants via videos throughout the recording period.

Continuous MEG signals were first denoised using the dual-signal subspace projection algorithm (37) in RICOH MEG Analysis. Dual-signal subspace projection is functionally comparable to the temporally extended signal space separation algorithm, differing mainly in the signal subspace projector approximation (38). Further signal processing was conducted using MNE-Python (39). Line-noise components (50 Hz and harmonics) were removed using MNE-Python’s spectrum_fit approach, which estimates and subtracts narrowband sinusoidal components in the frequency domain to reduce time-domain ringing artifacts that can emerge from applying multiple notch filters (40). Finally, signal-space projection (41) was applied to remove heartbeat (electrocardiography) and eye movement (electrooculography) artifacts. The detailed preprocessing code is provided in **Supplementary Material 2**.

### 2.4 Spatiotemporal cluster test

As no specific spatiotemporal hypothesis was formulated regarding ISM, data-driven analyses were conducted using the spatiotemporal cluster test (42) implemented in MNE-Python for sensor- and source-level data. We compared brain activity during the *Forward-order unmasking* with that during the *Reverse-order control* to investigate the differences in their spatiotemporal distributions. First, at each time point and sensor channel (or source vertex), a one-way analysis of variance was performed to compute the F-values by comparing the two ERFs. A threshold corresponding to *p* ≤ 0.01 was applied to the F-values, and spatially and temporally adjacent data points exceeding this threshold were grouped into clusters. The cluster statistic was defined as the sum of the F-values within each cluster. The significance of each cluster was assessed by comparing its statistics against the empirical distribution of the maximum cluster statistics obtained from 10,000 permutations. Clusters with a cluster-corrected *p* < 0.05 were considered statistically significant. The analysis time window for the spatiotemporal cluster test was set to 0–1000 ms relative to the onset of the face image. The same parameters were used for the sensor- and source-level analyses.

### 2.5 MEG sensor-level analysis

To minimize the edge effects associated with subsequent bandpass filtering, epochs were extracted over a wide time window of -2500 to 2500 ms relative to the onset of the face image. Baseline correction was applied using a -200 to 0 ms prestimulus interval. Epochs containing artifacts >4000 fT were excluded from further analyses. The remaining epochs were averaged to obtain ERFs, which were then filtered using a 1–25 Hz bandpass filter. The 1–25 Hz bandpass was selected to focus on low-frequency ERF components and obtain stable spatiotemporal cluster formation. Cluster stability checks across alternative upper cutoffs are provided in **Supplementary Material 3**. Subsequently, the ERFs were converted to Z-scores using the -200 to 0 ms baseline, and a spatiotemporal cluster test was performed to compare the waveforms between the *Forward-order unmasking* and *Reverse-order control*.

The difference in the waveform between the *Forward-order unmasking* and *Reverse-order control* was calculated for clusters identified as significant using the spatiotemporal cluster test. The mean difference waveform within the cluster was computed and defined as the ISM. This scalar summary measure was used for subsequent correlation analyses with clinical symptom scores in the SZ group. The difference waveform and the ISM are defined as follows:


$$D(t)=\;E_{Forward}(t)-\;E_{Reverse}(t)$$

$$ISM=\frac{1}{N}\sum_{i=1}^{N}D(t_{i})$$

Here, *E*_Forward_(*t*) and *E*_Reverse_(*t*) represent the ERFs for the *Forward-order unmasking* and *Reverse-order control*, respectively. D(t) denotes the difference waveform at each data point t., and N denotes the total number of data points within the cluster.

### 2.6 MEG source-level analysis

For MEG source estimation, structural magnetic resonance imaging scans were acquired for each participant at the Kumagaya General Hospital using a Discovery MR750w Expert 3.0T scanner. The T1-weighted images were obtained with a slice thickness of 1 mm. These T1-weighted images were individually reconstructed using the FreeSurfer software (43–45).

Data epoching and averaging were performed using the same procedure as that used for sensor-level analysis. Source estimation was then conducted on the averaged ERFs using MNE-Python (39).

Forward head modeling was performed using a single-compartment boundary-element model defined by the inner skull surface (46). The source current distribution was estimated using a noise-normalized dynamic statistical parametric mapping method (47, 48). The noise covariance matrix was computed from recordings in an empty room. To reduce the bias of the estimated source locations toward superficial currents, a depth-weighting parameter (depth = 0.8) was incorporated by adjusting the source covariance matrix (49). The resulting source time series were morphed onto the FreeSurfer standard brain template, “fsaverage” (50). To reduce the computational cost of the spatiotemporal cluster test, we adopted the “fsaverage” template with 1,284 vertices (51).

The source time courses were bandpass filtered between 1 and 25 Hz and then converted to Z-scores using the -200 to 0 ms baseline period. The spatiotemporal cluster test was conducted to compare the waveforms between the *Forward-order unmasking* and the *Reverse-order control*. The ISM values were computed using the same procedure as that used for sensor-level analysis.

### 2.7 Between-group comparisons

To test for group differences in the ISM, we performed a spatiotemporal cluster-based permutation test on the *Forward–Reverse* difference waveforms across sensors (or vertices) and time, using the same cluster-forming threshold and permutation scheme as in the within-group analyses. For each participant, the *Forward–Reverse* difference was computed at each sensor/vertex and time point, and these difference data were then compared between groups.

As a complementary analysis, we conducted a temporal cluster-based permutation test on the *Forward–Reverse* difference waveform averaged within a sensor/vertex ROI. This ROI was defined as the set of sensors/vertices comprising the significant within-group cluster (*Forward* vs. *Reverse*) observed in HCs for individual faces. As this ROI was defined based on the present dataset, the ROI-based temporal analysis should be interpreted as a follow-up analysis. The specific sensors/vertices included in the ROI are shown in **Supplementary Figure 1**.

### 2.8 Performance analysis

Behavioral performance (accuracy and response time) for the identity-judgment task was separately summarized for each participant, for each face type (individual and average), and for each trial type (*Forward-order unmasking* and *Reverse-order control*). In addition to descriptive statistics (mean ± SD), we determined main and interaction effects using a mixed-design ANOVA with Group (HC and SZ) as a between-subject factor and Face type (individual and average) and Order (*Forward-order unmasking* and *Reverse-order control*) as within-subject factors. Repeated measurements were accounted for by including Subject as a blocking factor in the model, and Type II sums of squares were used to obtain F-statistics. Effect sizes are reported as partial eta squared (ηp²).

We assessed normality of residuals using Shapiro–Wilk tests complemented by residual Q–Q plots, and we tested homogeneity of variance between groups using Levene’s tests within each Face type × Order cell (**Supplementary Tables 1B, C**; **Supplementary Figure 2**). Departures from normality were more apparent for response time (RT) residuals (particularly in HCs); therefore, we additionally reported analyses using log-transformed RT (**Supplementary Table 1D**).

### 2.9 Correlation analysis

Pearson’s correlation coefficients were computed to examine whether the sensor- and source-level results were associated with clinical symptom scales in the SZ group. As a significant cluster was identified only in HCs (individual face condition), we defined the spatiotemporal region of interest based on this cluster and then computed ISM within the same region of interest for participants with SZ. We tested whether ISM was correlated with the GAF and PANSS scores, antipsychotic medication dosage, and illness duration. To evaluate evidence for the absence of meaningful associations given the small sample size, we additionally performed equivalence tests using the two one-sided tests procedure on Fisher z-transformed correlation coefficients, with equivalence bounds set to ∣ρ∣ < 0.30.

## 3 Results

### 3.1 Performance data

**Table 2** provides a summary of the descriptive statistics (mean ± SD) for accuracy and response time in the identity-matching task (“Are the masked and unmasked faces of the same identity?”) across Group (HC and SZ), Face type (individual and average), and Order (*Forward-order unmasking* and *Reverse-order control*). Group-, Face type-, and Order-related effects were formally tested using a mixed 2 × 2 × 2 ANOVA (**Supplementary Table 1A**).

**Table 2:** Results of performance data.

			Accuracy		RT (s)	
Group	Face type	Order	Mean	SD	Mean	SD
HC	IND	*Forward-order*	0.898	0.130	0.903	0.463
HC	IND	*Reverse-order*	0.850	0.189	1.032	0.558
HC	AVG	*Forward-order*	0.828	0.074	0.974	0.688
HC	AVG	*Reverse-order*	0.757	0.186	1.211	0.818
SZ	IND	*Forward-order*	0.775	0.168	1.045	0.398
SZ	IND	*Reverse-order*	0.776	0.196	1.161	0.528
SZ	AVG	*Forward-order*	0.694	0.143	1.154	0.795
SZ	AVG	*Reverse-order*	0.693	0.159	1.292	0.577

Accuracy indicates the proportion of correct responses. RT denotes response time in seconds for each trial.

AVG, average face; HC, healthy control; IND, individual face; RT, response time; SZ, schizophrenia.

For accuracy, significant main effects of Group and Face type were observed, reflecting lower accuracy in the SZ group and in average faces than in individual faces. The main effect of Order was not significant. No two- or three-way interactions were observed.

For response time, significant main effects of Group, Face type, and Order were observed, reflecting slower responses in the SZ group, slower responses to average faces than to individual faces, and slower responses in the *Reverse-order control* than in the *Forward-order unmasking*. No two- or three-way interactions were observed. In a sensitivity analysis using log-transformed RTs to address departures from normality, the main effects of Group and Order remained significant, whereas the Face type effect was attenuated (**Supplementary Table 1D**).

### 3.2 Sensor-level analysis

**Figure 2:**
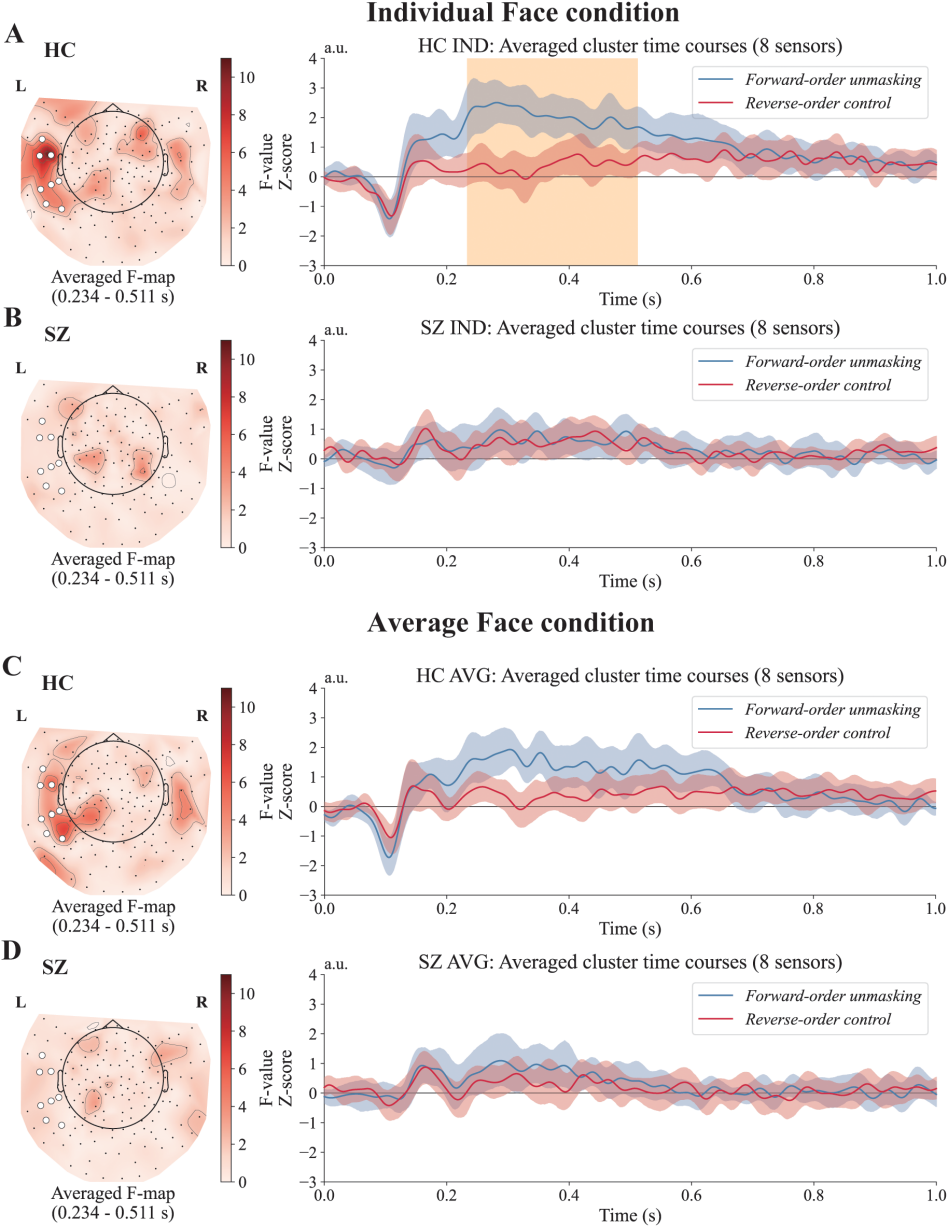
Results of the sensor-level analysis. Event-related fields (ERFs) for the contrast between *Forward-order unmasking* and *Reverse-order control* are shown separately for each group and face type. **(A)** HC group, individual faces (IND). A significant spatiotemporal cluster was observed across left temporal sensors between 234 and 511 ms, reflecting a difference between *Forward-order unmasking* and *Reverse-order control*. **(B)** SZ group, individual faces. No significant clusters were observed. **(C)** HC group, average faces (AVG). No significant clusters were observed. **(D)** SZ group, average faces. No significant clusters were observed. Scalp topographies indicate averaged F-maps during the 234–511 ms time window. Sensors highlighted in white indicate the clusters exhibiting a significant response in the HC group under the individual face condition. The blue line (*Forward-order unmasking*) shows the ERFs time-locked to mask removal. The red line (*Reverse-order control*) shows the ERFs time-locked to face presentation without the effect of mask removal. IND refers to individual face stimuli, and AVG refers to average face stimuli. The orange-shaded area indicates time points with cluster-corrected *p* < 0.05. The shaded areas around each ERF represent 95% confidence intervals.

**Figure 2** shows the results of the sensor-level analysis. For each group and face type, we compared *Forward-order unmasking* with *Reverse-order control* using a spatiotemporal cluster test. The orange-highlighted areas in the ERFs indicate the spatial and temporal windows, with the cluster-corrected *p* < 0.05. In the HC group, a significant difference was observed between 234 and 511 ms over the eight sensors in the left temporal region (indicated in white) when viewing individual faces in the *Forward-order unmasking* compared with the *Reverse-order control* (cluster mass ΣF = 14040.3, *p* = 0.046; peak F = 20.58 at 470 ms). No significant clusters were observed under other conditions. Therefore, we present the ERFs and mean F-map during this time window from the same eight sensors that showed significant responses in the individual face condition of the HC group. Full cluster statistics for all conditions are provided in **Supplementary Table 2**.

We next tested between-group differences in the *Forward–Reverse* difference (*Forward-order unmasking* minus *Reverse-order control*). As a primary whole-sensor analysis, we conducted a spatiotemporal cluster-based permutation test across sensors and time (0–1000 ms). This test did not yield a cluster surviving correction for either face type (best cluster: IND *p* = 0.169; AVG *p* = 0.757; **Supplementary Table 3A**).

**Figure 3:**
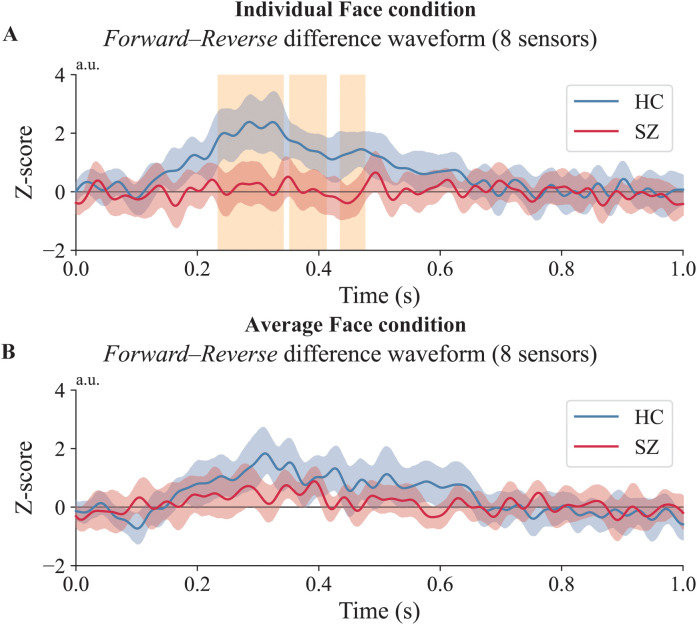
Results of the sensor-level between-group comparison. *Forward–Reverse* difference waveforms (*Forward-order unmasking* minus *Reverse-order control*) are shown separately for each face type, averaged across the eight left temporal sensors identified in the sensor-level analysis. **(A)** Individual faces. Significant temporal clusters were observed, reflecting a between-group difference (HC vs. SZ) in the *Forward–Reverse* difference waveform. **(B)** Average faces. No significant clusters were observed. The blue line (HC) and red line (SZ) show the *Forward–Reverse* difference waveforms. The orange-shaded area indicates time points with cluster-corrected *p* < 0.05. The shaded areas around each waveform represent 95% confidence intervals.

As a complementary follow-up analysis, we then performed an ROI-based temporal cluster test on the ROI-averaged *Forward–Reverse* difference waveform within the HC-defined sensor ROI (the eight left temporal sensors; **Supplementary Figure 1A**). In this follow-up ROI-based analysis, the *Forward–Reverse* difference waveform for individual faces was attenuated in individuals with SZ relative to HCs, with three significant temporal clusters (234–341 ms, *p* = 0.002; 352–412 ms, *p* = 0.011; 436–476 ms, *p* = 0.021). For average faces, no cluster survived correction (best cluster *p* = 0.111). Full ROI-based cluster statistics are provided in **Supplementary Table 3B**; illustrated in **Figure 3**.

### 3.3 Source-level analysis

**Figure 4:**
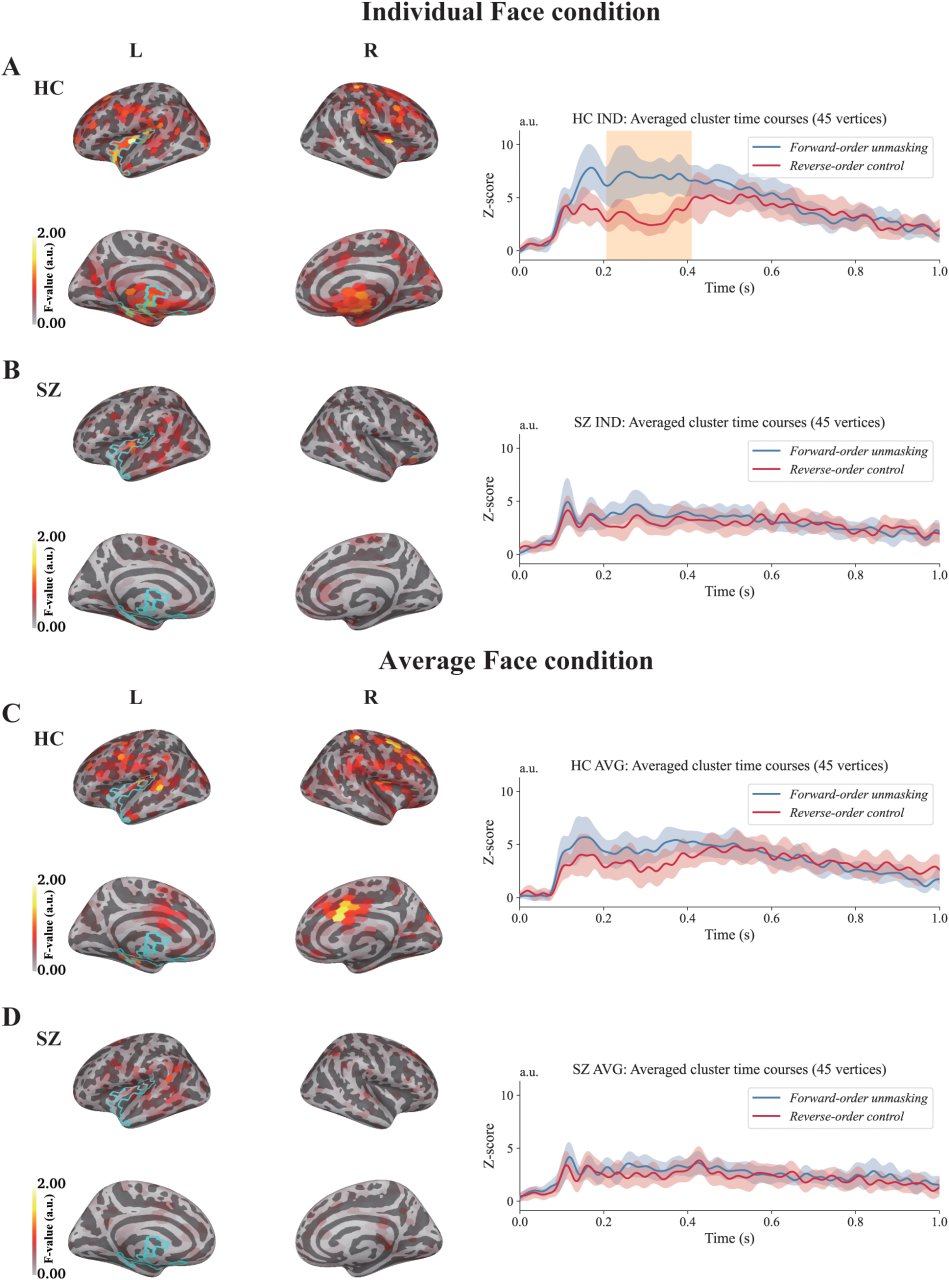
Results of the source-level analysis. **(A)** ERFs in the HC group when individual faces were presented. A significant response was observed in the cluster centered on the left insular cortex between 207 and 408 ms after mask removal. **(B)** ERFs in the HC group when average faces were presented. No significant responses were observed. **(C)** ERFs in the SZ group when individual faces were presented. No significant responses were observed. **(D)** ERFs in the SZ group when average faces were presented. No significant responses were observed. Brain images showing averaged F-maps during the 207–408 ms time window. The vertices outlined in cyan indicate significant clusters in the HC group under the individual face condition. The blue line (*Forward-order unmasking*) shows the ERFs time-locked to mask removal. The red line (*Reverse-order control*) shows the ERFs time-locked to face presentation without the effect of mask removal. IND denotes individual face stimuli, and AVG denotes average face stimuli. The orange highlighted area indicates time points, with cluster-corrected *p* < 0.05. The shaded areas around each ERF represent 95% confidence intervals.

**Figure 4** shows the results of the source-level analysis. For each group and face type, we compared the neural responses between the *Forward-order unmasking* and the *Reverse-order control* using a spatiotemporal cluster test. The orange-highlighted areas indicate the spatial and temporal windows, with cluster-corrected *p* < 0.05. In the HC group, a significant difference was observed between 207 and 408 ms in the vertices centered on the left insula (outlined by the cyan line) when viewing individual faces in the *Forward-order unmasking* compared with the *Reverse-order control* (cluster mass ΣF = 41202.0, *p* = 0.014; peak F = 23.88 at 353 ms). No significant clusters were observed under other conditions. Therefore, we present the ERFs and mean F-map during this time window from the same vertices that showed a significant response in the individual face condition of the HC group. Full cluster statistics for all conditions are provided in **Supplementary Table 4**.

For the between-group comparison of the *Forward–Reverse* difference at the source level, the primary whole-cortex spatiotemporal cluster-based test across vertices and time (0–1000 ms) did not identify any clusters surviving correction for either face type (best cluster: IND *p* = 0.104; AVG *p* = 0.776; **Supplementary Table 5A**).

**Figure 5:**
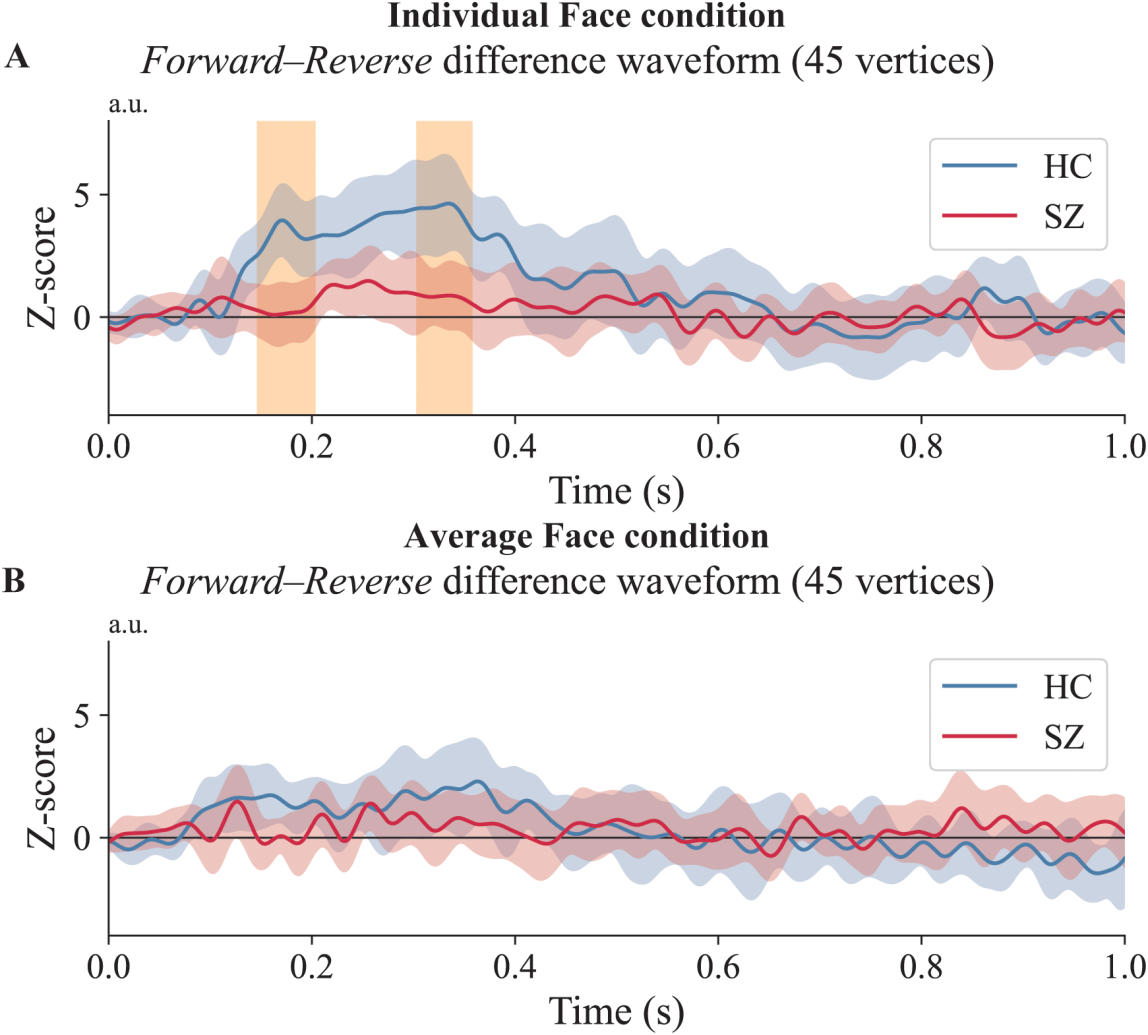
Results of the source-level between-group comparison. *Forward–Reverse* difference waveforms (*Forward-order unmasking* minus *Reverse-order control*) are shown separately for each face type, averaged across vertices within the cluster centered on the left insular cortex identified in the source-level analysis. **(A)** Individual faces. Significant temporal clusters were observed, reflecting a between-group difference (HC vs. SZ) in the *Forward–Reverse* difference waveform. **(B)** Average faces. No significant clusters were observed. The blue line (HC) and red line (SZ) show the *Forward–Reverse* difference waveforms. The orange-shaded area indicates time points with cluster-corrected p < 0.05. The shaded areas around each waveform represent 95% confidence intervals.

As a complementary follow-up analysis, we then performed an ROI-based temporal cluster test on the ROI-averaged *Forward–Reverse* difference waveform within the HC-defined insula-centered ROI (**Supplementary Figure 1B**). In this follow-up ROI-based test, the *Forward–Reverse* difference waveform was attenuated in the SZ group relative to the HC group for individual faces, with two significant temporal clusters (147–202 ms, *p* = 0.012; 304–357 ms, *p* = 0.020). For average faces, no cluster survived correction (best cluster *p* = 0.332). Full ROI-based cluster statistics are provided in **Supplementary Table 5B** and illustrated in **Figure 5**.

### 3.4 Correlations with clinical variables in SZ

In the SZ group, we examined Pearson correlations between the ISM for individual faces, defined as the mean difference wave (*Forward-order unmasking* minus *Reverse-order control*) extracted from the HC-defined significant clusters at the sensor and source levels, and clinical variables (GAF, PANSS scores, disease duration, and chlorpromazine-equivalent antipsychotic dose). No correlations remained significant after Benjamini–Hochberg false-discovery-rate correction across clinical variables at either level (**Supplementary Tables 6**, **7**).

Equivalence tests (two one-sided tests; equivalence bounds r = ± 0.30) provided no evidence that any correlation was practically equivalent to zero (all *p*_two one-sided tests ≥ 0.132), indicating that the data were inconclusive regarding the absence of small-to-moderate associations.

## 4 Discussion

In this study, brain responses in the SZ and HC groups were investigated following removing the mask from the individual and average faces. In HCs, mask removal from individual faces elicited a significant response in a cluster of sensors over the left temporal region, whereas no robust response was observed for average faces. Meanwhile, in the SZ group, no robust cluster-level mismatch response was detected in either face condition in the present dataset. For the between-group comparison of the *Forward–Reverse* difference, the primary whole-sensor (and whole-cortex) spatiotemporal cluster-based permutation tests did not identify any clusters surviving correction. However, in complementary ROI-based temporal cluster analyses aligned with the HC within-group clusters, the *Forward–Reverse* difference waveform for individual faces was attenuated in individuals with SZ relative to HCs at the sensor and source levels, whereas no robust effects were observed for average faces. Notably, the absence of a cluster surviving correction in the primary whole-sensor/whole-cortex analyses reflects the conservative nature of spatiotemporal cluster correction across a large search space, rather than providing strong evidence for or against the presence of a group-related effect. Therefore, the complementary ROI-based analyses were included to summarize whether group differences were expressed within the spatiotemporal region showing the most robust mismatch response in HCs. The timing of the significant effects in the sensor- and source-level analyses was approximately 200–500 ms after stimulus onset, overlapping with the MMN (12, 19, 20) and P300 component (52) time windows, likely reflecting predictive processing and attentional mechanisms, respectively. Considering the partial overlap with the MMN and P300 time windows, the observed responses may reflect processes following the typical N/M170 face-selective response associated with general face recognition (53–55).

In HCs, a significant neural response was observed only when the mask was removed from individual faces, but not from average faces. The facial images presented in the *Forward-order unmasking* and *Reverse-order control* were from the same unmasked faces; nonetheless, the priming effect of previously viewing the same masked face elicited a distinct neural response in the *Forward-order unmasking*. Thus, participants may have predicted the occluded facial regions using an internal average face model. Furthermore, the discrepancy between the predicted and actual facial features upon mask removal may lead to a mismatch response. This interpretation aligns with that of prior psychological studies showing that individuals tend to perceptually fill in masked facial regions based on an internal facial average (24) and that facial attractiveness tends to regress toward the average when masked (25, 26). Conversely, no robust response was observed in the SZ group during the *Forward-order unmasking*. This is consistent with previous findings of predictive processing disruption in SZ, evident by reductions in MMN (12). Particularly, this impairment has been reported in omission paradigms, in which an expected stimulus is omitted (56–58), and in the many-standards paradigm, in which multiple standard stimuli are presented (59). Notably, conventional MMN paradigms provide a well-established assay of mismatch processing based on violations of externally defined regularities, such as deviations from repetitive standards or omissions. Building on this framework, our paradigm complements these approaches by examining a related form of mismatch: the discrepancy between an internally inferred representation of an occluded face and the sensory evidence revealed upon unmasking—an ISM.

Notably, in this study, mismatch responses in HCs were observed in the absence of an explicit standard–deviant sequence or other externally defined regularities typical of conventional MMN paradigms. Unmasking individual faces elicited a reliable cluster-level mismatch response in the HC group, whereas the SZ group did not experience a robust cluster-level mismatch response. This pattern suggests that healthy individuals may rely on internal representations to infer occluded facial features under the task demands of masked face recognition, potentially drawing on templates from average faces. In the SZ group, the absence of a robust cluster-level mismatch response may reflect reduced precision of perceptual inference (60, 61). In addition, previous studies reporting attenuated N/M170 face-selective responses in SZ suggest that alterations in face-processing mechanisms may further contribute to reduced precision of face-related predictions (62, 63). Nevertheless, the observed group difference in ISM may be explained by reduced precision of perceptual inference and by potential differences between the SZ and HC groups in the facial representations used to complete occluded regions (i.e., the internal average-face template). In particular, previous studies have reported that individuals with SZ rely more on low-spatial-frequency information during facial-expression perception and use different diagnostic facial cues compared with HCs (64, 65). Therefore, a limitation of this study is that, owing to our design, we cannot clearly disentangle representational-template differences from differences in inferential precision. Future work should address this issue by combining the present paradigm with methods that directly estimate internal face templates, such as reverse correlation approaches (66).

Another important finding of this study was the involvement of the left insular cortex in the ISM observed in the HC group during mask removal. The insula potentially detects mismatches between sensory inputs and internal predictions and relays this information to the frontoparietal control network to facilitate attentional reallocation and cognitive control (67, 68). Notably, functional alterations of the insula have been reported in SZ (69, 70). Such alterations may reduce the ability to appropriately compare internally inferred representations with newly available sensory evidence in the present paradigm, thereby attenuating the mismatch response. Moreover, insular dysfunction has been discussed in relation to imprecise predictive coding in SZ (71), which could also contribute to lower precision of face-related predictions.

We further examined whether the ISM was associated with clinical symptom scales within the SZ group. Although significant associations with symptom scales were not observed from correlation analysis, this finding should not be interpreted as evidence of the absence of relationships. Consistent with this observation, equivalence testing did not support the conclusion that the associations were practically zero. Therefore, the present data are inconclusive regarding whether ISM are independent of clinical state or illness stage, and larger samples are required to clarify potential state- versus trait-like contributions.

To evaluate whether the observed neural effects could be explained by nonspecific differences in task performance or engagement, we examined behavioral accuracy and response times. The present findings could, in principle, be influenced by group differences in task performance or engagement. Behavioral analyses revealed a broadly consistent pattern across face types and orders: participants with SZ showed lower overall accuracy and slower response times than HCs. In the analysis of raw response times, responses also tended to be slower for average faces than for individual faces, and response times were slower in the *Reverse-order control* than in the *Forward-order unmasking*. To clarify, we found no clear evidence of an overall order effect on accuracy. Notably, no two- or three-way interactions were observed for either accuracy or response time. Accordingly, although generalized performance differences—particularly the greater difficulty of judging identity for less distinctive average faces—should be considered when interpreting the data, the absence of interactions suggests that reduced performance in SZ is not specific to a particular face type or presentation order. This pattern may partly reflect face-recognition difficulties in individuals with SZ reported in previous studies (72–74).

This study also has some limitations. First, a computational model that formally quantifies prediction precision, prediction error, or related Bayesian quantities was not included. Our model was designed as an exploratory study to detect neural responses to mask removal, but it lacks the parametric manipulations (e.g., varying mask opacity, familiarity levels, or prior probabilities) necessary for formal computational modeling. Future studies should incorporate (i) parametric manipulation of prediction strength, (ii) trial-by-trial measures of subjective uncertainty, (iii) repeated exposure to the same faces to enable learning-based computational models, and (iv) hierarchical Bayesian frameworks to estimate individual precision parameters. Accordingly, the term “ISM” is used here as a descriptive label for neural responses that may reflect prediction-related processing, rather than a computationally validated quantity. Second, all visual stimuli in the task involved Japanese female faces, and all participants were Japanese. The effects of racial or sex differences in the presented faces were not examined. Notably, the prediction of the masked facial region may be strongly influenced by an individual’s upbringing environment. Therefore, prediction precision may decrease for faces of unfamiliar races, leading to weaker ISM. Similarly, for familiar faces, deviations from the prediction may be small, producing weaker ISM, whereas unfamiliar faces of the same race can still elicit detectable ISM. Third, the average faces were generated from random combinations of 10 individuals, potentially resulting in insufficient control over the physical features of the individual and the average faces. Fourth, trial numbers differed across orders because of limitations in the available face-image set. Although this ensured the task feasibility and reduced participant burden, unequal trial counts may affect the signal-to-noise ratio and repetition effects. Future studies should expand the stimulus set and counterbalance order to better match trial counts between orders. Fifth, the sample size was relatively small, and patients with early-stage SZ were not included. Further studies are required to verify whether our findings apply to early-stage patients and clinically high-risk groups. Sixth, source-localization results should be interpreted cautiously because SZ is associated with structural brain changes that may affect forward/inverse modeling (75–77). Seventh, owing to the study design, we could not clearly disentangle and test group differences in the facial representations (templates) used to complete occluded regions from group differences in the precision of perceptual inference. Finally, the ROI-based between-group analyses were conducted as follow-up tests using ROIs defined from the HC within-group clusters in the same dataset, and thus the ROI was defined based on effects observed in the HC group within the same dataset and is therefore not statistically independent. Hence, these ROI-based findings should be considered complementary and require replication in an independent sample.

In conclusion, using MEG and a masked-face paradigm, we characterized an ISM, occurring when an internally inferred representation of an occluded face is updated by newly available sensory evidence. In HCs, unmasking individual faces evoked a reliable response over left temporal sensors, with source estimates suggesting left insula involvement. In the SZ group, no robust cluster-level mismatch response was detected, a pattern compatible with predictive-processing accounts implicating reduced precision of perceptual inference and consistent with the Hyperlearning Hypothesis. Future studies with larger samples and designs that more directly quantify precision are necessary to clarify underlying mechanisms, including contributions from face-processing.

## Data Availability

The original contributions presented in the study are included in the article/**Supplementary Material**. Further inquiries can be directed to the corresponding author.

## References

[1] Adams RA Stephan KE Brown HR Frith CD Friston KJ . The computational anatomy of psychosis. Front Psychiatry. (2013) 4:47. doi: 10.3389/fpsyt.2013.00047, PMID: 23750138 PMC3667557

[2] Friston K Brown HR Siemerkus J Stephan KE . The dysconnection hypothesis (2016). Schizophr Res. (2016) 176:83–94. doi: 10.1016/j.schres.2016.07.014, PMID: 27450778 PMC5147460

[3] Sterzer P Adams RA Fletcher P Frith C Lawrie SM Muckli L . The predictive coding account of psychosis. Biol Psychiatry. (2018) 84:634–43. doi: 10.1016/j.biopsych.2018.05.015, PMID: 30007575 PMC6169400

[4] Takei Y Sunaga M Fujihara K Ohki T Kato Y Jinde S . Hyperlearning Hypothesis: Network disruption and maladaptive learning in schizophrenia. Prog Neuropsychopharmacol Biol Psychiatry. (2026) 144:111599. doi: 10.1016/j.pnpbp.2025.111599, PMID: 41453483

[5] Hipólito I Kirchhoff M . Breaking boundaries: The Bayesian Brain Hypothesis for perception and prediction. Conscious. Cognit. (2023) 111:103510. doi: 10.1016/j.concog.2023.103510, PMID: 37058949

[6] Knill DC Pouget A . The Bayesian brain: The role of uncertainty in neural coding and computation. Trends Neurosci. (2004) 27:712–9. doi: 10.1016/j.tins.2004.10.007, PMID: 15541511

[7] Friston K . The history of the future of the Bayesian brain. NeuroImage. (2012) 62:1230–3. doi: 10.1016/j.neuroimage.2011.10.004, PMID: 22023743 PMC3480649

[8] Colombo M Seriès P . Bayes in the brain—On Bayesian modelling in neuroscience. Br J Philos Sci. (2012) 63:697–723. doi: 10.1093/bjps/axr043, PMID: 32691291

[9] Friston KJ Frith CD . Active inference, communication and hermeneutics. Cortex. J Devoted. Study. Nerv. Syst Behav. (2015) 68:129–43. doi: 10.1016/j.cortex.2015.03.025, PMID: 25957007 PMC4502445

[10] Goodwin I Kugel J Hester R Garrido MI . Bayesian accounts of perceptual decisions in the nonclinical continuum of psychosis: Greater imprecision in both top-down and bottom-up processes. PloS Comput Biol. (2023) 19:e1011670. doi: 10.1371/journal.pcbi.1011670, PMID: 37988398 PMC10697609

[11] Sundermeier M Standke I Schubotz RI Dannlowski U Lencer R Mecklenbrauck F . Alterations of task-based fMRI topology underlying cognitive flexibility and stability in schizophrenia. Neuroimage. (2025) 318:121416. doi: 10.1016/j.neuroimage.2025.121416, PMID: 40796019

[12] Kirihara K Tada M Koshiyama D Fujioka M Usui K Araki T . A predictive coding perspective on mismatch negativity impairment in schizophrenia. Front Psychiatry. (2020) 11:660. doi: 10.3389/fpsyt.2020.00660, PMID: 32733298 PMC7360815

[13] Casado-Román L Carbajal GV Pérez-González D Malmierca MS . Prediction error signaling explains neuronal mismatch responses in the medial prefrontal cortex. PloS Biol. (2020) 18:e3001019. doi: 10.1371/journal.pbio.3001019, PMID: 33347436 PMC7785337

[14] Stefanics GB Astikainen P Czigler IN . Visual mismatch negativity (vMMN): A prediction error signal in the visual modality. Front Hum Neurosci. (2015) 8:1074. doi: 10.3389/fnhum.2014.01074, PMID: 25657621 PMC4302941

[15] Garrido MI Kilner JM Stephan KE Friston KJ . The mismatch negativity: A review of underlying mechanisms. Clin Neurophysiol. (2009) 120:453–63. doi: 10.1016/j.clinph.2008.11.029, PMID: 19181570 PMC2671031

[16] Haenschel C Vernon DJ Dwivedi P Gruzelier JH Baldeweg T . Event-related brain potential correlates of human auditory sensory memory-trace formation. J Neurosci. (2005) 25:10494–501. doi: 10.1523/JNEUROSCI.1227-05.2005, PMID: 16280587 PMC6725828

[17] Baldeweg T Klugman A Gruzelier J Hirsch SR . Mismatch negativity potentials and cognitive impairment in schizophrenia. Schizophr Res. (2004) 69:203–17. doi: 10.1016/j.schres.2003.09.009, PMID: 15469194

[18] Winkler I . Interpreting the mismatch negativity. J Psychophysiol. (2007) 21:147–63. doi: 10.1027/0269-8803.21.34.147, PMID: 31409215

[19] Wacongne C . A predictive coding account of MMN reduction in schizophrenia. Biol Psychol. (2016) 116:68–74. doi: 10.1016/j.biopsycho.2015.10.011, PMID: 26582536

[20] Fong CY Law WHC Uka T Koike S . Auditory mismatch negativity under predictive coding framework and its role in psychotic disorders. Front Psychiatry. (2020) 11:557932. doi: 10.3389/fpsyt.2020.557932, PMID: 33132932 PMC7511529

[21] Tervaniemi M . Mismatch negativity–stimulation paradigms in past and in future. Front Neurosci. (2022) 16:1025763. doi: 10.3389/fnins.2022.1025763, PMID: 36466164 PMC9713013

[22] Fitzgerald K Todd J . Making sense of mismatch negativity. Front Psychiatry. (2020) 11:468. doi: 10.3389/fpsyt.2020.00468, PMID: 32595529 PMC7300203

[23] Pascalis O De Martin De Viviés X Anzures G Quinn PC Slater AM Tanaka JW . Development of face processing. Wiley. Interdiscip. Rev Cognit Sci. (2011) 2:666–75. doi: 10.1002/wcs.146, PMID: 22039564 PMC3203018

[24] Kramer RSS Jones AL . Incomplete faces are completed using a more average face. Cognit Res.: Princ. Implic. (2022) 7:79. doi: 10.1186/s41235-022-00429-y, PMID: 35984540 PMC9388992

[25] Pazhoohi F Kingstone A . Unattractive faces are more attractive when the bottom-half is masked, an effect that reverses when the top-half is concealed. Cognit Res.: Princ. Implic. (2022) 7:6. doi: 10.1186/s41235-022-00359-9, PMID: 35072804 PMC8785149

[26] Bassiri-Tehrani B Nguyen A Choudhary A Guart J Di Chiaro B Purnell CA . The effect of wearing a mask on facial attractiveness. Aesthet. Surg J Open Forum. (2022) 4:ojac070. doi: 10.1093/asjof/ojac070, PMID: 36320221 PMC9494328

[27] Trujillo LT Jankowitsch JM Langlois JH . Beauty is in the ease of the beholding: A neurophysiological test of the averageness theory of facial attractiveness. Cognit Affect Behav Neurosci. (2014) 14:1061–76. doi: 10.3758/s13415-013-0230-2, PMID: 24326966 PMC4053512

[28] Halit H de Haan M Johnson MH . Modulation of event-related potentials by prototypical and atypical faces. NeuroReport. (2000) 11:1871–5. doi: 10.1097/00001756-200006260-00014, PMID: 10884035

[29] Oldfield RC . The assessment and analysis of handedness: The Edinburgh inventory. Neuropsychologia. (1971) 9:97–113. doi: 10.1016/0028-3932(71)90067-4, PMID: 5146491

[30] First M JBW Karg RS Spitzer RL . Structured Clinical Interview for DSM-5—Research Version (SCID-5 for DSM-5, Research Version; SCID-5-RV). Arlington, Va: American Psychiatric Association (2015).

[31] Kay SR Fiszbein A Opler LA . The positive and negative syndrome scale (PANSS) for schizophrenia. Schizophr Bull. (1987) 13:261–76. doi: 10.1093/schbul/13.2.261, PMID: 3616518

[32] Matsuoka K . Development of Japanese Adult Reading Test (JART) for predicting premorbid IQ in mild dementia. Seishin. Igaku. (2002) 44:503–11.

[33] Hall RCW . Global assessment of functioning: A modified scale. Psychosomatics. (1995) 36:267–75. doi: 10.1016/S0033-3182(95)71666-8, PMID: 7638314

[34] Inada T Inagaki A . Psychotropic dose equivalence in Japan. Psychiatry Clin Neurosci. (2015) 69:440–7. doi: 10.1111/pcn.12275, PMID: 25601291

[35] Quek A . Facemorpher (2019). Available online at: https://github.com/alyssaq/facemorpher (Accessed October 18, 2022).

[36] DeBruine L . debruine/webmorph. (2018), Beta release 2. v0.0.0.9001. (Glasgow: Zenodo).

[37] Sekihara K Kawabata Y Ushio S Sumiya S Kawabata S Adachi Y . Dual signal subspace projection (DSSP): A novel algorithm for removing large interference in biomagnetic measurements. J Neural Eng. (2016) 13:36007. doi: 10.1088/1741-2560/13/3/036007, PMID: 27064933 PMC6287966

[38] Cai C Kang H Kirsch HE Mizuiri D Chen J Bhutada A . Comparison of DSSP and tSSS algorithms for removing artifacts from vagus nerve stimulators in magnetoencephalography data. J Neural Eng. (2019) 16:066045. doi: 10.1088/1741-2552/ab4065, PMID: 31476752

[39] Gramfort A Luessi M Larson E Engemann DA Strohmeier D Brodbeck C . MEG and EEG data analysis with MNE-Python. Front Neurosci. (2013) 7:267. doi: 10.3389/fnins.2013.00267, PMID: 24431986 PMC3872725

[40] Mitra P Bokil H . Observed Brain Dynamics. New York: Oxford University Press (2007). doi: 10.1093/acprof:oso/9780195178081.001.0001, PMID:

[41] Uusitalo MA Ilmoniemi RJ . Signal-space projection method for separating MEG or EEG into components. Med Biol Eng Comput. (1997) 35:135–40. doi: 10.1007/bf02534144, PMID: 9136207

[42] Maris E Oostenveld R . Nonparametric statistical testing of EEG- and MEG-data. J Neurosci Methods. (2007) 164:177–90. doi: 10.1016/j.jneumeth.2007.03.024, PMID: 17517438

[43] Dale AM Fischl B Sereno MI . Cortical surface-based analysis. I. Segmentation and surface reconstruction. Neuroimage. (1999) 9:179–94. doi: 10.1006/nimg.1998.0395, PMID: 9931268

[44] Fischl B Sereno MI Dale AM . Cortical surface-based analysis. II: Inflation, flattening, and a surface-based coordinate system. Neuroimage. (1999) 9:195–207. doi: 10.1006/nimg.1998.0396, PMID: 9931269

[45] Reuter M Schmansky NJ Rosas HD Fischl B . Within-subject template estimation for unbiased longitudinal image analysis. Neuroimage. (2012) 61:1402–18. doi: 10.1016/j.neuroimage.2012.02.084, PMID: 22430496 PMC3389460

[46] Hämäläinen MS Sarvas J . Realistic conductivity geometry model of the human head for interpretation of neuromagnetic data. IEEE Trans BioMed Eng. (1989) 36:165–71. doi: 10.1109/10.16463, PMID: 2917762

[47] Hashizume A Hironaga N . Principles of magnetoencephalography. In: Tobimatsu S Kakigi R , editors. Clinical Applications of Magnetoencephalography. Springer, Tokyo (2016). p. 3–32. doi: 10.1007/978-4-431-55729-6_1, PMID:

[48] Hayamizu M Hagiwara K Hironaga N Ogata K Hoka S Tobimatsu S . A spatiotemporal signature of cortical pain relief by tactile stimulation: An MEG study. Neuroimage. (2016) 130:175–83. doi: 10.1016/j.neuroimage.2016.01.065, PMID: 26854558

[49] Lin FH Witzel T Ahlfors SP Stufflebeam SM Belliveau JW Hämäläinen MS . Assessing and improving the spatial accuracy in MEG source localization by depth-weighted minimum-norm estimates. Neuroimage. (2006) 31:160–71. doi: 10.1016/j.neuroimage.2005.11.054, PMID: 16520063

[50] Greve DN van der Haegen L Cai Q Stufflebeam S Sabuncu MR Fischl B . A surface-based analysis of language lateralization and cortical asymmetry. J Cognit Neurosci. (2013) 25:1477–92. doi: 10.1162/jocn_a_00405, PMID: 23701459 PMC3767398

[51] Fischl B Sereno MI Tootell RBH Dale AM . High-resolution intersubject averaging and a coordinate system for the cortical surface. Hum Brain Mapp. (1999) 8:272–84. doi: 10.1002/(sici)1097-0193(1999)8:4<272::aid-hbm10>3.0.co;2-4 PMC6873338 10619420

[52] Polich J . Updating P300: An integrative theory of P3a and P3b. Clin Neurophysiol. (2007) 118:2128–48. doi: 10.1016/j.clinph.2007.04.019, PMID: 17573239 PMC2715154

[53] Xu Y Liu J Kanwisher N . The M170 is selective for faces, not for expertise. Neuropsychologia. (2005) 43:588–97. doi: 10.1016/j.neuropsychologia.2004.07.016, PMID: 15716149

[54] Murashko AA Shmukler A . EEG correlates of face recognition in patients with schizophrenia spectrum disorders: A systematic review. Clin Neurophysiol. (2019) 130:986–96. doi: 10.1016/j.clinph.2019.03.027, PMID: 31003117

[55] Bentin S Allison T Puce A Perez E Mccarthy G . Electrophysiological studies of face perception in humans. J Cognit Neurosci. (1996) 8:551–65. doi: 10.1162/jocn.1996.8.6.551, PMID: 20740065 PMC2927138

[56] Rudolph ED Ells EML Campbell DJ Abriel SC Tibbo PG Salisbury DF . Finding the missing-stimulus mismatch negativity (MMN) in early psychosis: Altered MMN to violations of an auditory gestalt. Schizophr Res. (2015) 166:158–63. doi: 10.1016/j.schres.2015.05.028, PMID: 26072323 PMC4791035

[57] Kreitschmann-Andermahr I Rosburg T Meier T Volz HP Nowak H Sauer H . Impaired sensory processing in male patients with schizophrenia—A magnetoencephalographic study of auditory mismatch detection. Schizophr Res. (1999) 35:121–9. doi: 10.1016/S0920-9964(98)00115-7, PMID: 9988849

[58] Yabe H Tervaniemi M Reinikainen K Näätänen R . Temporal window of integration revealed by MMN to sound omission. NeuroReport. (1997) 8:1971–4. doi: 10.1097/00001756-199705260-00035, PMID: 9223087

[59] Koshiyama D Kirihara K Tada M Nagai T Fujioka M Usui K . Reduced auditory mismatch negativity reflects impaired deviance detection in schizophrenia. Schizophr Bull. (2020) 46:937–46. doi: 10.1093/schbul/sbaa006, PMID: 32072183 PMC7345817

[60] Edwards MJ Adams RA Brown H Pareés I Friston KJ . A Bayesian account of ‘hysteria’. Brain. (2012) 135:3495–512. doi: 10.1093/brain/aws129, PMID: 22641838 PMC3501967

[61] Zahedi A Lynn SJ Sommer W . Cognitive simulation along with neural adaptation explain effects of suggestions: A novel theoretical framework. Front Psychol. (2024) 15:1388347. doi: 10.3389/fpsyg.2024.1388347, PMID: 38966744 PMC11223671

[62] Ohara N Hirano Y Oribe N Tamura S Nakamura I Hirano S . Neurophysiological face processing deficits in patients with chronic schizophrenia: An MEG study. Front Psychiatry. (2020) 11:554844. doi: 10.3389/fpsyt.2020.554844, PMID: 33101080 PMC7495506

[63] Onitsuka T Oribe N Nakamura I Kanba S . Review of neurophysiological findings in patients with schizophrenia. Psychiatry Clin Neurosci. (2013) 67:461–70. doi: 10.1111/pcn.12090, PMID: 24102977

[64] Laprévote V Oliva A Delerue C Thomas P Boucart M . Patients with schizophrenia are biased toward low spatial frequency to decode facial expression at a glance. Neuropsychologia. (2010) 48:4164–8. doi: 10.1016/j.neuropsychologia.2010.10.017, PMID: 20955721

[65] Faghel-Soubeyrand S Lecomte T Bravo MA Lepage M Potvin S Abdel-Baki A . Abnormal visual representations associated with confusion of perceived facial expression in schizophrenia with social anxiety disorder. NPJ Schizophr. (2020) 6:28. doi: 10.1038/s41537-020-00116-1, PMID: 33004809 PMC7529755

[66] Brinkman L Dotsch R Zondergeld J Koevoets MGJC Aarts H Van Haren NEM . Visualizing mental representations in schizophrenia patients: A reverse correlation approach. Schizophr Res Cognit. (2019) 17:100138. doi: 10.1016/j.scog.2019.100138, PMID: 31008060 PMC6454059

[67] Menon V . Salience network. Brain Mapp. (2015) 2:597–611. doi: 10.1016/B978-0-12-397025-1.00052-X, PMID: 41810140

[68] Menon V Uddin LQ . Saliency, switching, attention and control: A network model of insula function. Brain Struct Funct. (2010) 214:655–67. doi: 10.1007/s00429-010-0262-0, PMID: 20512370 PMC2899886

[69] Kittleson AR Woodward ND Heckers S Sheffield JM . The insula: Leveraging cellular and systems-level research to better understand its roles in health and schizophrenia. Neurosci Biobehav Rev. (2024) 160:105643. doi: 10.1016/j.neubiorev.2024.105643, PMID: 38531518 PMC11796093

[70] Wylie KP Tregellas JR . The role of the insula in schizophrenia. Schizophr Res. (2010) 123:93–104. doi: 10.1016/j.schres.2010.08.027, PMID: 20832997 PMC2957503

[71] Liddle PF Liddle EB . Imprecise predictive coding is at the core of classical schizophrenia. Front Hum Neurosci. (2022) 16:818711. doi: 10.3389/fnhum.2022.818711, PMID: 35308615 PMC8928728

[72] Bortolon C Capdevielle D Raffard S . Face recognition in schizophrenia disorder: A comprehensive review of behavioral, neuroimaging and neurophysiological studies. Neurosci Biobehav Rev. (2015) 53:79–107. doi: 10.1016/j.neubiorev.2015.03.006, PMID: 25800172

[73] Chen Y Ekstrom T . Visual and associated affective processing of face information in schizophrenia: A selective review. Curr Psychiatry Rev. (2015) 11:266–72. doi: 10.2174/1573400511666150930000817, PMID: 27134614 PMC4849484

[74] Marwick K Hall J . Social cognition in schizophrenia: A review of face processing. Br Med Bull. (2008) 88:43–58. doi: 10.1093/bmb/ldn035, PMID: 18812413

[75] Gupta CN Calhoun VD Rachakonda S Chen J Patel V Liu J . Patterns of gray matter abnormalities in schizophrenia based on an international mega-analysis. Schizophr Bull. (2015) 41:1133–42. doi: 10.1093/schbul/sbu177, PMID: 25548384 PMC4535628

[76] Haijma SV Van Haren N Cahn W Koolschijn PCMP Hulshoff Pol HE Kahn RS . Brain volumes in schizophrenia: A meta-analysis in over 18–000 subjects. Schizophr Bull. (2013) 39:1129–38. doi: 10.1093/schbul/sbs118, PMID: 23042112 PMC3756785

[77] Herkströter F Zahedi A Standke I Dannlowski U Lencer R Schubotz RI . Gray matter matters: Cognitive stability and flexibility in schizophrenia spectrum disorder. Psychophysiology. (2024) 61:e14596. doi: 10.1111/psyp.14596, PMID: 38691383

