# Supplementary material for: Attenuated inferred–sensory mismatch during masked face recognition in schizophrenia
Source: Front Psychiatry. 2026 Mar 16;17:1749435. doi: 10.3389/fpsyt.2026.1749435 (PMC13034134; doi:10.3389/fpsyt.2026.1749435)
Supplement: Supplementary file 1 [file DataSheet1.pdf]

Supplementary Material

1 Supplementary Tables and Figures

1.1 Supplementary Tables

Supplementary Table 1a. ANOVA results for behavioral performance

DV	Effect	df1	df2	F	*p*-vaue	ηp²
Accuracy	Group	1	102	11.04	0.001*	0.098
Accuracy	Face type	1	102	25.56	0.000*	0.200
Accuracy	Order	1	102	3.41	0.068	0.032
Accuracy	Group × Face type	1	102	0.00	0.989	0.000
Accuracy	Group × Order	1	102	3.31	0.072	0.031
Accuracy	Face type × Order	1	102	0.15	0.700	0.001
Accuracy	Group × Face type × Order	1	102	0.09	0.764	0.001
RT (s)	Group	1	102	9.04	0.003*	0.081
RT (s)	Face type	1	102	4.17	0.044*	0.039
RT (s)	Order	1	102	6.68	0.011*	0.061
RT (s)	Group × Face type	1	102	0.00	0.965	0.000
RT (s)	Group × Order	1	102	0.22	0.641	0.002
RT (s)	Face type × Order	1	102	0.29	0.592	0.003
RT (s)	Group × Face type × Order	1	102	0.13	0.722	0.001

*Statistical significance: $p < 0.05$

Face type: IND (individual faces), AVG (average faces). Order: *Forward-order unmasking*, *Reverse-order control*. RT was calculated as the mean across trials for each combination of Face type and Order for each participant. Values shown as 0.000 indicate $p < 0.001$.

ANOVA, analysis of variance; DV, dependent variable; RT, response time; ηp², partial eta squared. Group: HC (healthy control), SZ (schizophrenia)

Supplementary Table 1b Shapiro–Wilk tests of normality for ANOVA residuals

DV	Group	Face type	Order	W	*p*-value
Accuracy	HC	IND	*Forward-order*	0.904	0.067
Accuracy	HC	IND	*Reverse-order*	0.883	0.029*
Accuracy	HC	AVG	*Forward-order*	0.849	0.008*
Accuracy	HC	AVG	*Reverse-order*	0.848	0.008*
Accuracy	SZ	IND	*Forward-order*	0.963	0.662
Accuracy	SZ	IND	*Reverse-order*	0.958	0.557
Accuracy	SZ	AVG	*Forward-order*	0.973	0.843
Accuracy	SZ	AVG	*Reverse-order*	0.939	0.277
RT	HC	IND	*Forward-order*	0.835	0.005*
RT	HC	IND	*Reverse-order*	0.756	0.000*
RT	HC	AVG	*Forward-order*	0.509	0.000*
RT	HC	AVG	*Reverse-order*	0.685	0.000*
RT	SZ	IND	*Forward-order*	0.947	0.381
RT	SZ	IND	*Reverse-order*	0.986	0.992
RT	SZ	AVG	*Forward-order*	0.784	0.001*
RT	SZ	AVG	*Reverse-order*	0.966	0.728
logRT	HC	IND	*Forward-order*	0.889	0.037*
logRT	HC	IND	*Reverse-order*	0.794	0.001*
logRT	HC	AVG	*Forward-order*	0.684	0.000*
logRT	HC	AVG	*Reverse-order*	0.947	0.378
logRT	SZ	IND	*Forward-order*	0.899	0.055
logRT	SZ	IND	*Reverse-order*	0.980	0.953
logRT	SZ	AVG	*Forward-order*	0.933	0.218
logRT	SZ	AVG	*Reverse-order*	0.932	0.212

*Statistical significance: $p < 0.05$

Note: Shapiro–Wilk tests were applied to residuals from the mixed ANOVA reported in Supplementary Table 1a. Values shown as 0.000 indicate $p < 0.001$. Although Shapiro–Wilk tests indicated some non-normality for Accuracy residuals, Q–Q plots suggested only minor departures (Supplementary Figure 1); deviations were most evident for RT residuals in the HC group, motivating the additional analysis on log-transformed RT (Supplementary Table 1d).

ANOVA, analysis of variance; W, Shapiro–Wilk statistic; RT, response time; logRT, log-transformed RT; AVG, average faces; HC, healthy control; IND, individual faces; SZ, schizophrenia

Supplementary Table 1c. Levene's tests of homogeneity of variance on ANOVA residuals

DV	Face type	Order	W	*p*-value
Accuracy	IND	*Forward-order*	0.244	0.624
Accuracy	IND	*Reverse-order*	0.000	0.991
Accuracy	AVG	*Forward-order*	0.237	0.630
Accuracy	AVG	*Reverse-order*	0.011	0.916
RT	IND	*Forward-order*	0.154	0.697
RT	IND	*Reverse-order*	0.115	0.736
RT	AVG	*Forward-order*	0.051	0.823
RT	AVG	*Reverse-order*	0.166	0.686
logRT	IND	*Forward-order*	0.345	0.561
logRT	IND	*Reverse-order*	0.040	0.842
logRT	AVG	*Forward-order*	0.280	0.600
logRT	AVG	*Reverse-order*	0.015	0.904

Note: Levene's tests were applied to residuals from the mixed ANOVA (Supplementary Table 1a) to assess homogeneity of variance between groups within each Face type × Order cell. All tests were nonsignificant ($p > 0.05$), indicating no evidence for heteroscedasticity.
ANOVA, analysis of variance; W, Levene statistic; RT, response time; logRT, log-transformed RT; AVG, average faces; IND, individual faces

Supplementary Table 1d. ANOVA results on log(RT)

DV	Effect	df1	df2	F	*p*-value	ηp^2
log(RT)	Group	1	102	19.46	0.000*	0.160
log(RT)	Face type	1	102	1.75	0.189	0.017
log(RT)	Order	1	102	13.39	0.000*	0.116
log(RT)	Group × Face type	1	102	0.00	0.991	0.000
log(RT)	Group × Order	1	102	0.42	0.520	0.004
log(RT)	Face type × Order	1	102	1.92	0.169	0.018
log(RT)	Group × Face type × Order	1	102	0.04	0.834	0.000

*Statistical significance: $p < 0.05$

Note: To address non-normality in RT residuals (Supplementary Table 1b), we additionally conducted the same mixed ANOVA on log-transformed RT [log(RT)]. Values shown as 0.000 indicate $p < 0.001$. In this analysis, the main effects of Group and Order remained significant, whereas the Face type effect and all interactions were not significant.

ANOVA, analysis of variance; RT, response time; log(RT), log-transformed RT; ηp^2, partial eta squared

Supplementary Table 2. Best spatiotemporal cluster statistics in the sensor-level analysis.

Group	Face type	Cluster *p* (corr)	Time window (ms)	Sensors (n)	Cluster mass (ΣF)	Peak F	Peak time (ms)
HC	IND	0.046	234–511	8	14040.3	20.58	470
SZ	IND	0.426	831–872	10	3238.5	15.28	854
HC	AVG	0.112	896–971	10	8267.1	21.04	916
SZ	AVG	0.815	902–978	1	1593.4	11.32	920

Note: For each group and face type, we performed a spatiotemporal cluster-based permutation test comparing the *Forward-order unmasking* and *Reverse-order control*. For each test, we report only the "best cluster," defined as the cluster with the smallest cluster-level *p*-value among all detected clusters, regardless of statistical significance. Reported *p*-values are cluster-level, permutation-corrected.
AVG, average faces; HC, healthy control; IND, individual faces; SZ, schizophrenia

Supplementary Table 3a. Between-group spatiotemporal cluster test on the *Forward–Reverse* difference waveform (sensor level; best cluster only)

Face type	Cluster *p* (corr)	Time window (ms)	Cluster mass (ΣF)	Peak F	Peak time (ms)
IND	0.169	266–426	9876.0	20.05	320
AVG	0.757	186–246	3506.9	11.77	209

Note: For each face type, we compared HC and SZ groups using a spatiotemporal cluster-based permutation test on the sensor-level *Forward–Reverse* difference data (*Forward-order unmasking* minus *Reverse-order control*) across sensors and time (0–1000 ms). Reported *p*-values are cluster-level, permutation-corrected. For completeness, we report the best (minimum-*p*) cluster for each face type, regardless of statistical significance. Cluster mass indicates ΣF (the sum of F-values within the cluster). Peak F and peak time indicate the maximum F-value within the cluster and its latency.
AVG, average faces; HC, healthy control; IND, individual faces; SZ, schizophrenia

Supplementary Table 3b. Between-group ROI-based temporal cluster test on the *Forward–Reverse* difference waveform (sensor level)

Face type	Cluster *p* (corr)	Time window (ms)	Cluster mass (ΣF)	Peak F	Peak time (ms)
IND	0.002	234–341	2552.0	16.84	324
IND	0.011	352–412	1242.6	14.97	370
IND	0.021	436–476	935.4	13.62	459
AVG	0.111	307–326	378.2	11.41	317

Note: For each face type, we compared HC and SZ groups using a temporal cluster-based permutation test on the sensor-level *Forward–Reverse* difference waveform (*Forward-order unmasking* minus *Reverse-order control*) averaged within the sensor ROI. Reported *p*-values are cluster-level, permutation-corrected. We report all clusters with cluster-level $p < 0.05$; when no cluster survived correction, we additionally report the best (minimum-*p*) cluster for completeness. Cluster mass indicates ΣF (the sum of F-values within the cluster). Peak F and peak time indicate the maximum F-value within the cluster and its latency.

AVG, average faces; HC, healthy control; IND, individual faces; SZ, schizophrenia

Supplementary Table 4. Best spatiotemporal cluster statistics in the source-level analysis.

Group	Face type	Cluster p (corr)	Time window (ms)	Vertices (n)	Cluster mass (ΣF)	Peak F	Peak time (ms)
HC	IND	0.014	207–408	45	41202	23.88	353
SZ	IND	0.922	547–598	6	2467.6	11.98	589
HC	AVG	0.188	339–431	11	11461.3	25.07	352
SZ	AVG	0.344	674–742	13	6336.2	16.11	712

Note: For each group and face type, we performed a spatiotemporal cluster-based permutation test comparing the *Forward-order unmasking* and *Reverse-order control*. For each test, we report only the "best cluster," defined as the cluster with the smallest cluster-level p-value among all detected clusters, regardless of statistical significance. Reported p-values are cluster-level, permutation-corrected.
AVG, average faces; HC, healthy control; IND, individual faces; SZ, schizophrenia

Supplementary Table 5a. Between-group spatiotemporal cluster test on the *Forward–Reverse* difference waveform (source level; best cluster only)

Face type	Cluster *p* (corr)	Time window (ms)	Cluster mass (ΣF)	Peak F	Peak time (ms)
IND	0.104	380–466	29063.1	22.74	433
AVG	0.776	696–773	8924.3	21.95	744

Note: For each face type, we compared HC and SZ groups using a spatiotemporal cluster-based permutation test on the source-level *Forward–Reverse* difference data (*Forward-order unmasking* minus *Reverse-order control*) across vertices and time (0–1000 ms). Reported *p*-values are cluster-level, permutation-corrected. For completeness, we report the best (minimum-*p*) cluster for each face type, regardless of statistical significance. Cluster mass indicates ΣF (the sum of F-values within the cluster). Peak F and peak time indicate the maximum F-value within the cluster and its latency.
AVG, average faces; HC, healthy control; IND, individual faces; SZ, schizophrenia

Supplementary Table 5b. Between-group ROI-based temporal cluster test on the *Forward–Reverse* difference waveform (source level)

Face type	Cluster p (corr)	Time window (ms)	Cluster mass (ΣF)	Peak F	Peak time (ms)
IND	0.012	147–202	1283.3	15.19	168
IND	0.020	304–357	1019.5	10.55	344
AVG	0.332	976–981	76.2	7.69	979

Note: For each face type, we compared HC and SZ groups using a temporal cluster-based permutation test on the source-level *Forward–Reverse* difference waveform (*Forward-order unmasking* minus *Reverse-order control*) averaged within the source ROI. Reported p-values are cluster-level, permutation-corrected. We report all clusters with cluster-level $p < 0.05$; when no cluster survived correction, we additionally report the best (minimum-p) cluster for completeness. Cluster mass indicates ΣF (the sum of F-values within the cluster). Peak F and peak time indicate the maximum F-value within the cluster and its latency.

AVG, average faces; HC, healthy control; IND, individual faces; SZ, schizophrenia

Supplementary Table 6. Correlations between sensor-level ISM and clinical variables in SZ.

Clinical variable	r	*p*-value	*p*_FDR	*p*_TOST	Equiv.
GAF	0.087	0.732	0.878	0.195	No
PANSS positive	0.320	0.196	0.513	0.534	No
PANSS negative	0.021	0.933	0.933	0.132	No
PANSS general	0.273	0.273	0.513	0.455	No
Disease duration	0.238	0.342	0.513	0.397	No
Antipsychotic (chlorpromazine equivalent dose mg/day)	0.478	0.045	0.269	0.793	No

Note: Sensor-level ISM was defined as the mean difference waveform (*Forward-order unmasking* minus *Reverse-order control*) for individual faces, extracted from the HC-defined significant sensor-level cluster. Pearson's r is shown. *p*_FDR indicates Benjamini–Hochberg FDR correction across clinical variables. Equivalence was assessed using TOST with equivalence bounds r = ±0.30; *p*_TOST is the overall TOST *p*-value.

ISM, inferred–sensory mismatch; GAF, Global Assessment of Functioning; PANSS, Positive and Negative Syndrome Scale

Supplementary Table 7. Correlations between source-level ISM and clinical variables in SZ.

Clinical variable	r	*p*-value	*p*_FDR	*p*_TOST	Equiv.
GAF	-0.314	0.205	0.307	0.523	No
PANSS positive	0.586	0.011	0.063	0.92	No
PANSS negative	0.439	0.068	0.137	0.734	No
PANSS general	0.445	0.064	0.137	0.744	No
Disease duration	-0.145	0.565	0.565	0.264	No
Antipsychotic (chlorpromazine equivalent dose mg/day)	-0.166	0.51	0.565	0.292	No

Note: Sensor-level ISM was defined as the mean difference waveform (*Forward-order unmasking* minus *Reverse-order control*) for individual faces, extracted from the HC-defined significant source-level cluster. Pearson's r is shown. *p*_FDR indicates Benjamini–Hochberg FDR correction across clinical variables. Equivalence was assessed using TOST with equivalence bounds r = ±0.30; *p*_TOST is the overall TOST *p*-value.
ISM, inferred–sensory mismatch; GAF, Global Assessment of Functioning; PANSS, Positive and Negative Syndrome Scale

1.2 Supplementary Figures

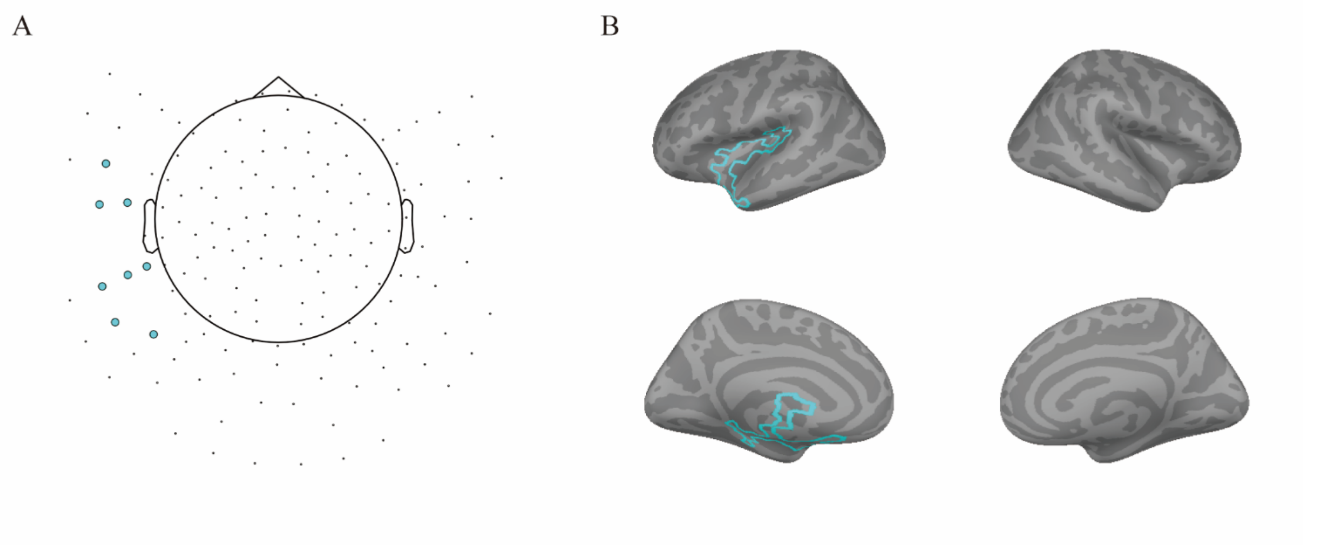

Supplementary Figure 1. Sensor- and source-level ROIs used for the complementary ROI-based temporal cluster test. (**A**) Sensor-level ROI. Sensors included in the ROI are highlighted in cyan on the sensor layout (all sensors shown as black dots). The ROI included the following sensor indices and channel names: 39 (LT33), 40 (LT34), 42 (LT24), 53 (LT36), 54 (LT37), 56 (LT26), 57 (LT28), and 59 (LT16). (**B**) Source-level ROI. The ROI on the fsaverage cortical surface is outlined in cyan (lateral and medial views). The ROI vertices are provided as FreeSurfer vertex indices on the fsaverage source space. In this ROI, all vertices were located in the left hemisphere (lh) and none in the right hemisphere (rh). The lh vertex indices were: 27, 38, 77–78, 80–82, 125–126, 144, 147, 161, 200–201, 269–270, 273, 275, 280, 282, 325–326, 334, 358, 395, 428–429, 431, 434–435, 536–539, 589–590, 598, 600–601, 611, 634–635, 639–641.

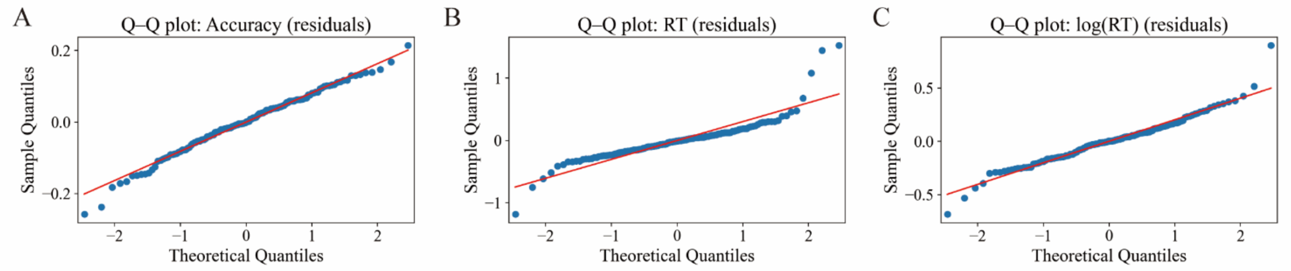

Supplementary Figure 2. Residual Q–Q plots for ANOVA. Q–Q plots show ANOVA residuals (A: Accuracy; B: RT; C: log(RT)). Residuals for accuracy showed small departures from normality in some cells (**A**). RT residuals showed mild deviations from normality (**B**), which were attenuated after log transformation (**C**). ANOVA, analysis of variance; RT, response time; log(RT), log-transformed RT

1.3 Supplementary Data

Supplementary Material 1. Exclusion criteria

Participants were excluded if they met any of the following criteria:

1. Currently hospitalized (inpatient status).
2. Aged <18 years.
3. Contraindications to magnetic resonance imaging and/or magnetoencephalography, such as an implanted cardiac pacemaker or severe claustrophobia.
4. Severe physical comorbidity, defined as the presence of any of the following medical conditions:
    - Respiratory diseases (e.g., pneumonia, acute asthma attack, emphysema, acute exacerbation of interstitial pneumonia, pulmonary embolism, and pneumothorax).
    - Cardiac diseases (e.g., heart failure equivalent to New York Heart Association class III–IV, ischemic heart disease, and arrhythmia requiring monitoring).
    - Fractures requiring surgery or direct/indirect traction.
    - Spinal cord injury.
    - Severe endocrine/metabolic diseases (e.g., diabetes requiring insulin, endocrine disorders requiring specialist care, and hyperammonemia due to cirrhosis).
    - Severe malnutrition (e.g., eating disorder with body mass index < 15).
    - Disturbance of consciousness (e.g., acute drug intoxication, alcohol-related disorder, electrolyte imbalance, and delirium due to metabolic disease).
    - Systemic infections (e.g., tuberculosis, acquired immunodeficiency syndrome, primary/secondary syphilis, and sepsis).
    - Central nervous system infections (e.g., meningitis and encephalitis).
    - Acute abdomen (e.g., gastrointestinal bleeding and ileus).
    - Fulminant hepatitis or severe acute pancreatitis.
    - Neuroleptic malignant syndrome or rhabdomyolysis.
    - Extensive burns (involving at least half of an extremity).
    - Conditions requiring surgery, chemotherapy, or radiotherapy, or terminal malignancy.
    - Initiation of dialysis.
    - Severe hematologic diseases (e.g., anemia with hemoglobin ≤ 7 g/dL or requiring frequent transfusions).
    - Acute and severe renal diseases (e.g., acute renal failure, nephrotic syndrome, and glomerulonephritis).
    - Any condition requiring an operating-room procedure.
    - Collagen vascular diseases requiring specialist management.
    - Pregnancy or the postpartum period.
    - Designated intractable diseases (as defined by national regulations; certified cases).

Supplementary Material 2. Preprocessing code

```
from __future__ import annotations

from typing import List, Optional, Iterable
import numpy as np
import mne


def _infer_channels_by_type_or_name(raw, *, ch_type: str, name_keywords: List[str]) -> List[str]:
    ch_names = raw.info["ch_names"]
    picks = mne.pick_types(raw.info, **{ch_type: True})
    if len(picks) > 0:
        return [ch_names[i] for i in picks]
    out = []
    for name in ch_names:
        up = name.upper()
        if any(k in up for k in name_keywords):
            out.append(name)
    return out


def apply_notch_harmonics_and_ssp_ecg_eog(
    raw: mne.io.BaseRaw,
    *,
    line_freq: float = 50.0,
    max_notch_freq: float = 300.0,
    notch_widths: float | Iterable[float] = 4.0,
    notch_method: str = "spectrum_fit",
    n_jobs: int = 1,
    # ECG SSP
    ecg_ch_names: Optional[List[str]] = None,
    n_ecg_grad: int = 1,
    n_ecg_mag: int = 2,
    n_ecg_eeg: int = 0,
    # EOG SSP
    eog_ch_names: Optional[List[str]] = None,
    n_eog_grad: int = 1,
    n_eog_mag: int = 2,
    n_eog_eeg: int = 0,
    reject: Optional[dict] = None,
    verbose: Optional[str] = None,
) -> mne.io.BaseRaw:
    """
    Notch filter line-noise harmonics (e.g., 50,100,...,300 Hz) and apply SSP (ECG+EOG).
    """
    if not raw.preload:
        raise RuntimeError("raw must be preloaded (read_raw_fif(..., preload=True)).")
```

```
    raw_out = raw.copy()

    # 1) Notch filter: harmonics from 50 to 300 Hz
    freqs = np.arange(line_freq, max_notch_freq + 0.1, line_freq)  # 50,100,...,300
    raw_out.notch_filter(
        freqs=freqs,
        method=notch_method,      # spectrum_fit tends to be gentler than FIR notches
        notch_widths=notch_widths,
        n_jobs=n_jobs,
        verbose=verbose,
    )

    # 2) ECG SSP
    if ecg_ch_names is None:
        ecg_ch_names = _infer_channels_by_type_or_name(raw_out, ch_type="ecg",
name_keywords=["ECG", "EKG"])

    ecg_projs = []
    if len(ecg_ch_names) > 0:
        for ch in ecg_ch_names:
            projs, _ = mne.preprocessing.compute_proj_ecg(
                raw_out, ch_name=ch,
                n_grad=n_ecg_grad, n_mag=n_ecg_mag, n_eeg=n_ecg_eeg,
                reject=reject, n_jobs=n_jobs, verbose=verbose
            )
            ecg_projs.extend(projs)
    else:
        mne.utils.logger.warn("No ECG channel found. Skipping ECG-based SSP.")

    # 3) EOG SSP
    if eog_ch_names is None:
        eog_ch_names = _infer_channels_by_type_or_name(raw_out, ch_type="eog",
name_keywords=["EOG"])

    eog_projs = []
    if len(eog_ch_names) > 0:
        for ch in eog_ch_names:
            projs, _ = mne.preprocessing.compute_proj_eog(
                raw_out, ch_name=ch,
                n_grad=n_eog_grad, n_mag=n_eog_mag, n_eeg=n_eog_eeg,
                reject=reject, n_jobs=n_jobs, verbose=verbose
            )
            eog_projs.extend(projs)
    else:
        mne.utils.logger.warn("No EOG channel found. Skipping EOG-based SSP.")

    # 4) Apply SSP
    all_projs = ecg_projs + eog_projs
    if len(all_projs) > 0:
```

```
    raw_out.add_proj(all_projs)
    raw_out.apply_proj()
else:
    mne.utils.logger.warn("No SSP projectors were created; returning notch-only Raw.")

return raw_out
```

Supplementary Material 3. Cluster stability checks

	1–25 Hz		1–40 Hz		1–80 Hz	
	Mean	SD	Mean	SD	Mean	SD
Number of clusters (n)	48	-	75	-	130	-
Cluster duration (ms)	24.3	39.6	16.3	21.5	9.8	14.0
Cluster mass (Σ|F|)	997.8	2249.2	685.1	1331.9	391.3	945.4

Note: Cluster stability checks were performed by repeating the same spatiotemporal cluster-based permutation test with three band-pass filter settings (1–25, 1–40, and 1–80 Hz). "Number of clusters" indicates the count of spatiotemporal clusters identified in the observed statistic map at the cluster-forming threshold (i.e., prior to cluster-level correction). "Cluster duration" denotes the temporal extent of each cluster (ms). "Cluster mass" is defined as Σ|F|, the sum of absolute F-values within each cluster. Values are descriptive summaries (mean ± SD) across all clusters detected under each filtering setting, regardless of statistical significance.
